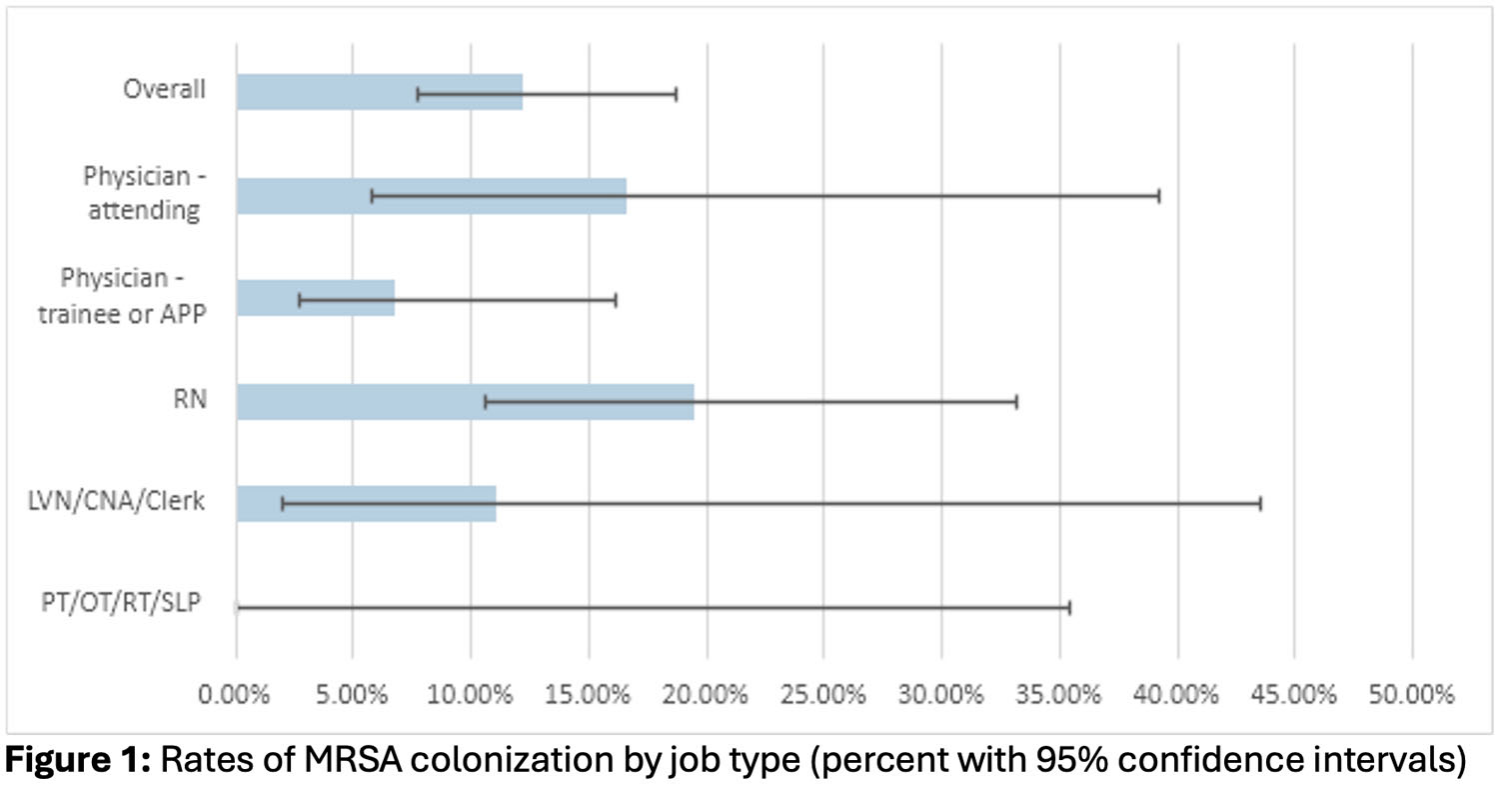# 313 Risk Factors for Progression to Invasive Candida Auris Infection among Colonized Patients

**DOI:** 10.1017/ash.2026.10664

**Published:** 2026-06-23

**Authors:** Curtis Sera, Niki Arab, Arthur Jeng, Sherif Shoucri

**Affiliations:** 1 Olive View-UCLA Medical Center

## Abstract

**Background:** Contact precautions are often utilized by hospital systems to prevent the spread of Methicillin-Resistant Staphylococcus aureus (MRSA). Increasingly, however, the effectiveness of these measures is being debated. While MRSA colonization rates have been studied in various patient populations, published healthcare worker (HCW) colonization rates vary. We therefore sought to quantify the rate of MRSA colonization among HCWs at our institution while surveying HCWs about their use of contact precautions. **Methods:** HCWs with direct patient care responsibilities were recruited to complete an anonymous survey about contact precaution adherence, followed by a swab of the bilateral anterior nares. Swabs were performed using an ESwab sample collection system and plated on MRSA selective media (HardyCHROM MRSA chromogenic). Cultures were interpreted within 48 hours of incubation. **Results:** A total of 139 HCWs completed the survey, and 132 swabs were collected. The majority of participants were physician trainees or advanced practice providers (43.2%), followed by nurses (34.1%) and attending physicians (11.4 %). The remainder were collected from a combination of nursing assistants and patient therapists. MRSA was detected in 17 HCWs (12.2%). Nurses represented the majority of positive cases (9/17). Compared with MRSA negative HCWs, MRSA-colonized HCWs had higher average years of work experience (10.2 [IQR 2.5–16.0] vs 8.7 [IQR 1.5–12.0]; p = 0.56) though it was not statistically significant. Most HCWs (87%) reported performing recommended hand hygiene more than 90% of the time. Among HCWs who tested positive for MRSA colonization, 9/17 (52.9%) predicted they would test positive MRSA, while 52/115 (45.2%) of HCWs who ultimately tested negative predicted they were colonized. Discussion: Our results showed that MRSA colonization rates among HCWs at our facility were similar to published rates at other North American institutions. Although further statistical evaluation is needed, it is noteworthy that MRSA-positive HCWs had worked in hospitals longer than MRSA-negative HCWs and that nurses had a disproportionate colonization rate. This suggests that time at the bedside may be a strong risk factor for MRSA colonization among HCWs. While the data supporting the use of contact precautions to prevent MRSA transmission are debated, the importance of hand hygiene is not. Reassuringly, 87% of HCWs reported on this anonymous survey that they performed hand hygiene more than 90% of the time. We aim to further analyze our data and evaluate how HCW colonization rates change as our MRSA contact isolation policies are updated in the future.

**Figure:**